# Impact of Globalization and Animal Trade on Infectious Disease Ecology

**DOI:** 10.3201/eid1312.071276

**Published:** 2007-12

**Authors:** Nina Marano, Paul M. Arguin, Marguerite Pappaioanou

**Affiliations:** * Centers for Disease Control and Prevention, Atlanta, Georgia, USA; † Association of American Veterinary Colleges, Washington, DC, USA

**Keywords:** zoonotic disease, animal importation, globalization, infectious diseases, introduction

**Figure:**
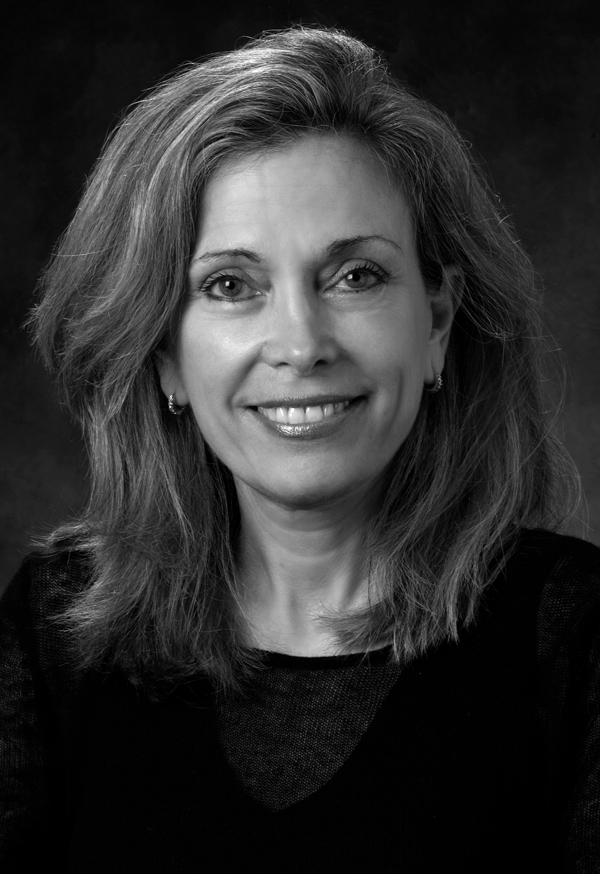
**Dr Marano** is chief of the Geographic Medicine and Health Promotion Branch in the Division of Global Migration and Quarantine, CDC. The Branch’s mission is to protect the health of international travelers and prevent the introduction of zoonotic diseases into the country through imported animals and animal products.

**Figure:**
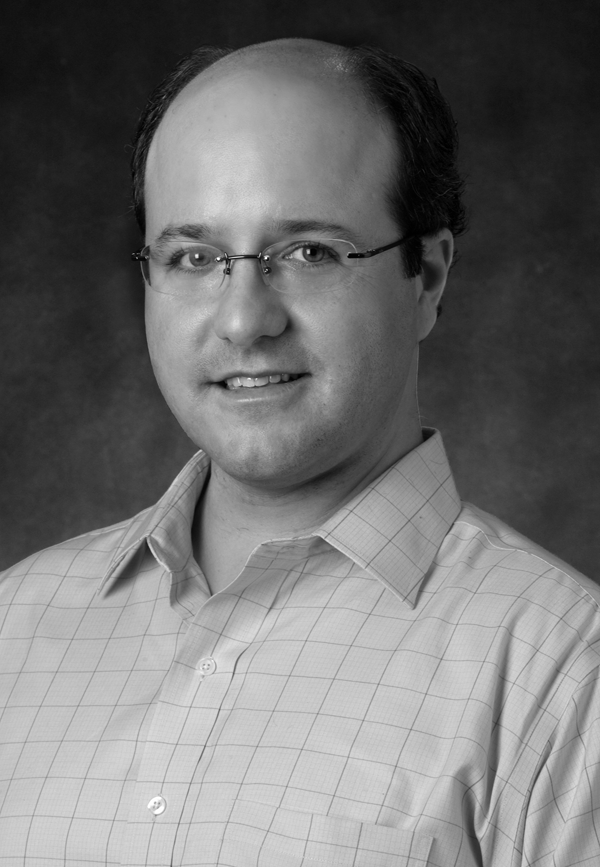
**Dr Arguin** is the chief of the Domestic Response Unit in the Malaria Branch within the National Center for Zoonotic, Vectorborne, and Enteric Diseases at CDC. His research interests include the prevention and treatment of infectious diseases associated with international travel, including malaria and zoonoses.

**Figure:**
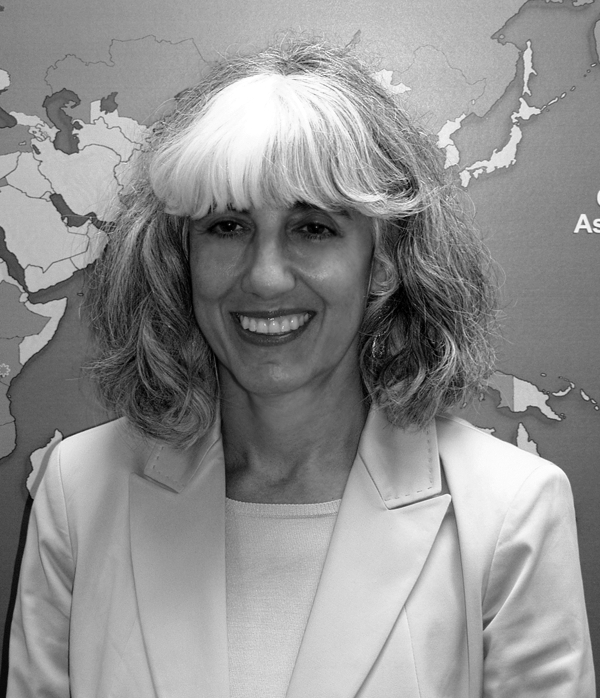
**Dr Pappaioanou** is executive director of the Association of American Veterinary Colleges in Washington, DC. Her areas of interest are in emerging zoonotic infectious diseases, with a special interest in influenza viruses and in collaborative efforts that bridge public health and domestic animal and wildlife health sectors that address emerging zoonotic infectious diseases.

The articles on rabies (*1*) and Marburg (*2*) virus featured in this month’s Emerging Infectious Diseases (EID) zoonoses issue illustrate common themes. Both discuss zoonotic diseases with serious health implications for humans, and both have a common reservoir, the bat. These articles, and the excitement generated by this year’s recognition of World Rabies Day on September 8, also described in this issue (*3*), remind us how globalization has had an impact on the worldwide animal trade. This worldwide movement of animals has increased the potential for the translocation of zoonotic diseases, which pose serious risks to human and animal health (*3*).

The magnitude of the global movement of animals is staggering. In terms of sheer numbers, 37,858,179 individually counted live amphibians, birds, mammals, and reptiles were legally imported to the United States from 163 countries in 2000–2004. These imports included Asian macaques, South American rodents, and African great cats (*4*).

Why do we have a global trade in animals? Animals are legally imported into the United States for many reasons. They are used for exhibitions at zoos; scientific education, research, and conservation programs; food and products; and in the case of companion animals, tourism and immigration. Increasingly, however, animals are being imported for a thriving commercial pet trade. In many cases the animals that are imported and traded are of species that are considered exotic (here defined as non-native species, animals not traditionally kept as pets, or both). This can be a risky business, as many shipments include a high volume of wild-caught versus captive raised. animals For most of these animals, there are no requirements for zoonotic disease screening either before or after arrival into the United States. There have been anecdotal reports of high rates of death among animals in these shipments.

Animals imported for commercial trade represent a substantial risk to human health. In 2003, monkeypox was introduced into the United States when a shipment of African Gambian giant rats was sold to dealers, one of whom housed the rats with prairie dogs intended for the pet trade in a US distribution facility. The prairie dogs subsequently became ill and transmitted the infection to 71 humans, including prairie dog owners and veterinary staff caring for the ill animals (*5*). In addition to monkeypox, human tularemia and salmonellosis outbreaks have been traced back to contact with prairie dogs and hedgehogs (*6*,*7*).

Exotic pet ownership brings unanticipated risks to agribusiness, wildlife conservation, and the ecosystem. For example, giant African land snails released into farmlands have become agricultural pests. They reproduce rapidly, consume large amounts of vegetation, and are hosts for parasites such as *Angiostrongylus cantonensis.* Pet pythons released into wetlands become unchecked predators, warping the balance of the existing food chain. Snakehead fish, imported as a delicacy for the live food markets, have turned up in ponds and waterways, where they quickly dominate the ecosystem at the expense of native species. And the illegal trade of exotic wildlife, with promises of considerable financial return in the underground markets, has disastrous implications for many endangered or threatened species.

How easy is it to get an exotic animal? Checked the Internet lately? It’s now possible to obtain almost any type of exotic pet animal through the Internet, as opposed to purchasing them in pet stores, which are subject to licensure and inspection.

As a scientist, one might suggest solutions that employ familiar tools, such as postarrival screening of animals with reliable laboratory tests, empirical treatment for known diseases (if such tests and treatments already existed), or quarantine of the animals for an appropriate length of time. Many of these solutions are not feasible or practical to use on the large volume of animals that are being imported and cannot be employed to prevent new or emerging pathogens or infections. Ultimately, import restrictions may be the only means of preventing introduction of exotic infections.

Despite the societal costs of importing exotic animals, as well as the difficulties in regulating enforcement and coordination of efforts, there are also benefits and compelling reasons for importing certain species of wildlife. Many wildlife conservation and species survival programs depend on importing exotic animals, including endangered species kept by zoos. Much is learned from captive wild animals and the knowledge gained about how to manage disease problems. Zoologic societies’ ability to use animals that are legally imported has enabled public education about endangered animals; were it not for legal animal importation and exhibition in zoological institutions, many species of animals, including bats, would be extinct in the wild.

Partnerships comprising experts and agencies involved with human, animal, and ecosystem health are critical to prevent and control imported zoonotic diseases. Such partnerships benefit public, animal, and ecosystem health. There are several unfortunate examples of the failure of partners across these areas to work together. They include governmental decisions in the People’s Republic of China to slaughter dogs as a control measure for rabies and advocating extermination of storks as a control measure for avian influenza in Thailand (*8*,*9*) In the case of the dogs, an integrated team of animal and public health professionals might have implemented alternate control measures, such as leash laws and rabies vaccination of dogs. The storks were luckier: wildlife conservationists and other partners in the animal health sector eventually intervened to convince governmental authorities that slaughter of storks was not an appropriate control measure for avian influenza.

As in the past 3 EID zoonoses theme issues, we have called for renewed effort for the public health and animal sectors to work together, in this case to mitigate the impact on infectious disease ecology caused by unrestricted translocation of animals. Prevention efforts should include reducing both the supply of and the demand for exotic animals. However, navigating the myriad responsibilities of the different sectors for human, livestock, companion animal and wildlife health continues to be a challenge. Guidelines addressing the infectious disease risks associated with exotic animals that may help raise awareness of the risks and decrease the demand for exotic animals have been published (*10*). However, no single agency can solve this problem alone; it is only through partnership with other federal agencies, wildlife associations, veterinary medical associations and private industry that we will be able to better control the global movement of animals and reduce the risk of introducing emerging infectious diseases into new locations.

The “One Medicine Initiative” announced by Roger Mahr, the 2006 President of the American Veterinary Medical Association, has led to the 2007 formation in the United States of a “One Health Task Force” to bring wildlife, environmental, human, and domestic animal sectors together for a coordinated approach to improving and protecting human and animal health (*11*). This coordinated approach, actively supported by multiple stakeholders, takes into account the larger ecologic context of infectious diseases and improves our ability to prevent disease rather than simply reacting to new outbreaks as they emerge. We look forward to the work of the Task Force and other important cross-disciplinary initiatives, as well as the efforts of the informed readership of EID to make important contributions in stemming the magnitude of live animal trade that poses risks to human, animal, and ecosystem health.
